# Clinico-radiological comparison and short-term prognosis of single acute pancreatitis and recurrent acute pancreatitis including pancreatic volumetry

**DOI:** 10.1371/journal.pone.0206062

**Published:** 2018-10-25

**Authors:** Maxim Avanesov, Anastassia Löser, Alla Smagarynska, Sarah Keller, Helena Guerreiro, Enver Tahir, Murat Karul, Gerhard Adam, Jin Yamamura

**Affiliations:** 1 Department of Diagnostic and Interventional Radiology and Nuclear Medicine, University Medical Center Hamburg-Eppendorf, Hamburg, Germany; 2 Department of Radiotherapy and Radiation Oncology, University Medical Center Hamburg-Eppendorf, Hamburg, Germany; 3 Department of Diagnostic and Interventional Radiology, Marienkrankenhaus, Hamburg, Germany; University of Nebraska Medical Center, UNITED STATES

## Abstract

**Purpose:**

The necrosis-fibrosis hypothesis describes a continuum between single attacks of acute pancreatitis (SAP), recurrent acute pancreatitis (RAP) and chronic pancreatitis (CP) with endocrine and exocrine pancreatic insufficiency. For prevention purposes we evaluated clinico-radiological parameters and pancreatic volumetry to compare SAP and RAP and provide prognostic relevance on short-term mortality, need for intervention and the hospitalization duration.

**Materials and methods:**

We retrospectively investigated 225 consecutive patients (150 males, range 19-97years) with acute pancreatitis (74%SAP, 26%RAP) according to the revised Atlanta classification. All patients received an intravenous contrast-enhanced CT after a median time of 5 (IQR 5–7) days after onset of symptoms. Two experienced observers rated the severity of AP by 3 CT scores (CTSI, mCTSI, EPIC). Moreover, total pancreatic volumes and additional parenchymal necrosis volumes were assessed, when appropriate. Clinical parameters were etiology of AP, lipase on admission, CRP 48 hours after admission (CRP48), and the presence of organ dysfunction, assessed by the modified Marshall score. The modified Marshall score included systolic blood pressure, serum creatinine, and the ratio of arterial oxygen partial pressure to fractional inspired oxygen (PaO_2_/FiO_2_ ratio) and was assessed on admission and 48 hours after admission to find patients with persistent organ failure. Outcome parameters were total hospitalization duration, short-term mortality and need for intervention.

**Results:**

Lipase, CRP48, etiology of AP, EPIC, PaO_2_/FiO_2_ ratio, and the presence of a pleural effusion differed significantly in both groups (p<0.05). In 109 patients with interstitial edematous AP, the total pancreatic volume was significantly smaller in patients with RAP compared to those with SAP (69±35cm^3^; (RAP) vs 106±45cm^3^; (SAP), p<0.001). All outcome parameters including the mortality rates (SAP vs. RAP: 15% vs. 7%) were comparable in both groups (p>0.05). In the necrotizing RAP group, only the necrotic volume correlated significantly with total hospitalization time (r = 0.72, p<0.001), whereas the systolic blood pressure was the only, but weak predictor for short-term mortality (β-coefficient: -0.05, p = 0.03) and the need for intervention (β-coefficient: -0.02, p = 0.048) in the total RAP group. In patients with SAP, the modified Marshall score was the strongest predictor of short-term mortality, followed by the mCTSI on multivariate logistic regression (Marshall score: β-coefficient: 1.79, p<0.001; mCTSI: β-coefficient: 0.40, p<0.001). CTSI was the best predictor for required intervention in necrotizing SAP (β-coefficient: 0.46, p<0.001), followed by the volume of intrapancreatic necrosis (β-coefficient: 0.17, p = 0.03).

**Conclusion:**

Total pancreatic volume differed significantly between interstitial RAP and SAP and intrapancreatic necrosis volume revealed prognostic value for the total hospitalization duration in necrotizing RAP. Although all outcome parameters were comparable between SAP and RAP, only systolic blood pressure and pancreatic volumetry were prognostic in RAP. In SAP, only the modified Marshall score and mCTSI revealed prognostic value for short-term mortality, whereas CTSI was predictive for the need for intervention.

## Introduction

Acute pancreatitis (AP) represents one of the most commonly found gastrointestinal diseases with an incidence of 4.6–100 per 100,000 cases in Europe [1] and a mortality ranging from 2–30% in case of persistent organ failure and necrotizing pancreatitis [2, 3]. According to the necrosis-fibrosis hypothesis [4], a continuum exists between single attacks of acute pancreatitis (SAP), recurrent acute pancreatitis (RAP) and chronic pancreatitis (CP) with persistent endocrine and exocrine pancreatic insufficiency [5]. RAP is thereby defined as two or more separate attacks of AP with complete resolution within more than three months in between the attacks [6] and was shown to evolve into CP in 4% to 24% of cases [7,8]. In contrast to CP, where manifest alterations of the pancreatic duct, evidence of parenchymal calcifications, fibrosis, and atrophy are seen [9], morphologic changes in RAP are poorly described and were not investigated by cross sectional imaging according to criteria proposed by the revised Atlanta classification [10]. Moreover, an accurate and objective differentiation between patients with SAP and RAP is crucial as there is growing evidence for possible reduction strategies for recurrent attacks of AP [11–15], thereby possibly preventing a chronification of the disease with irreversible pancreatic damage. Unfortunately, RAP is often underdiagnosed in up to 30% of cases [16] and the patients’ history of RAP may not be well documented or biased by language barriers as well as cultural or social reservations, especially in case of alcoholic RAP.

Recently, Meyrignac et al. reported about the additional value of the volumetric extent of extrapancreatic necrosis (EN) for prediction of pancreatitis outcome compared with established clinical and radiological parameters in patients with SAP [17]. However, its calculation in daily practice is limited because EN represents a separate entity with better outcome compared with pancreatic parenchymal necrosis [18], the diagnosis of EN is difficult due to a significant inter-observer variation [19] and its calculation is very time consuming [17].

Therefore, the aims of this study were 1) to assess clinico-radiological parameters to compare patients with SAP and RAP and to predict mortality, need for intervention, and hospitalization duration in both groups and 2) to evaluate the additional value of the volumetric extent of pancreatic parenchyma and parenchymal necrosis for characterization and short-term prognosis in SAP and RAP.

## Materials and methods

### Patient population

The study group included 225 consecutive patients (150 males, range 19–97 years) with AP according to the criteria of the revised Atlanta classification [10]. All patients received an intravenous contrast-enhanced abdominal CT between January 2011 and May 2017. The local Clinical Institutional Review Board (Ethic committee of the medical chamber of Hamburg) approved our retrospective single-centre study and waived the requirement for informed consent due to the anonymous analysis of all retrospective data.

All selected patients met the following inclusion and exclusion criteria:

**Inclusion criteria**:

1. AP according to the revised Atlanta classification [10]. Episodes of recurrent acute pancreatitis (RAP) were diagnosed with equal revised Atlanta criteria as for a single attack of acute pancreatitis (SAP).
2. Intravenous contrast-enhanced abdominal CT in the portal venous phase with evidence of AP.
3. Data available for analysis in our radiological information system (RIS).

**Exclusion criteria**:

1. Pregnancy.
2. Age below 18 years.
3. Less than 72 h delay between onset of abdominal symptoms and CT to accurately discriminate an interstitial edematous AP from necrotizing AP [20].
4. Patients with clinical and/or morphologic signs of manifest chronic pancreatitis [9]

### CT acquisition

CT was performed in all patients after a median time of 5 (IQR 5–7) days after onset of symptoms using a 256-detector row CT scanner (Brilliance iCT; Philips Medical Systems, Best, The Netherlands). All patients received 100 ml iomeprol (Imeron 300: Bracco, Konstanz, Germany) followed by a 20 ml saline chaser intravenously. None of the patients received enteric (oral or rectal) contrast material. Breath-hold imaging of the portal venous phase started 85 s after i.v. contrast agent application to enable a proper discrimination between edematous and necrotic pancreatitis and a sufficient assessment of the amount of pancreatic and extrapancreatic changes, as recommended and previously demonstrated [21,22]. The scan region covered the area from the diaphragm to the pubic symphysis. Technical parameters included the following: tube voltage: 120 kV, tube current: 80–160 mAs, axial field of view 350 mm, no gap, collimation 2 mm×128 mm× 0.625 mm, pitch 0.985, and gantry rotation time 0.33 s. The radiation dose was adjusted according to the patients’ body size and body shape by automatic current selection (ACR; Dose-Right; Philips Healthcare, Best, The Netherlands). Axial, sagittal, and coronal images were reconstructed from isotropic voxels with a slice thickness of 3 mm.

### Image analysis

All CT studies were retrospectively observed by two experienced radiologists with 34 years (AS) and 6 (MA) of experience in abdominal imaging. These two observers initially reviewed all available CT data of patients with AP and confirmed sufficient diagnostic examination quality in a consensus evaluation before inclusion of patients in the final study group. Both observers were blinded to the clinical data and outcome of the investigated patients. These two observers independently assessed signs of AP and categorized each case into one of the two morphologic groups: interstitial edematous pancreatitis and necrotizing pancreatitis according to the presence of the following findings:

Edematous organ swelling, peripancreatic fat stranding and/or fluid collection for the diagnosis of interstitial edematous pancreatitis [10,20] and parenchymal and/or peripancreatic necrosis, defined by absent pancreatic parenchymal contrast enhancement and/or heterogeneous fluid collection containing both liquefied and solid necrotic material [10,20] for the diagnosis of necrotizing acute pancreatitis. Thereby, heterogeneous fluid collection was defined by a density within the fluid of more than 20 hounsfield units (HU). In case of disagreement between the two observers regarding the morphologic type of AP, consensus was reached on a final reading by a third senior expert reader with 16 years of experience in abdominal imaging (JY).

### Pancreatitis severity score calculation

The degree of pancreatic and peripancreatic inflammation was obtained by the established CT severity index (CTSI) developed by Balthazar et al. [23] and the modified CT severity index (mCTSI) developed by Mortele et al. [24]. For extrapancreatic inflammatory changes, extrapancreatic inflammation on CT score (EPIC) developed by De Waele et al. [25] was calculated. The details of the scoring systems are described elsewhere [23–25]. Each score was applied independently to the investigated CT studies by both observers, who were blinded to all clinical parameters and to the scoring results of their counterpart. According to previously published data [25,26], the following cutoffs were used for predicting clinically severe disease: CTSI ≥4, mCTSI ≥6, and EPIC ≥3. Further statistical analysis of CTSI, mCTSI, and EPIC was based on the mean score values of the two abovementioned observers.

### Volumetry of pancreatic tissue and intrapancreatic necrosis

**Fig 1:**
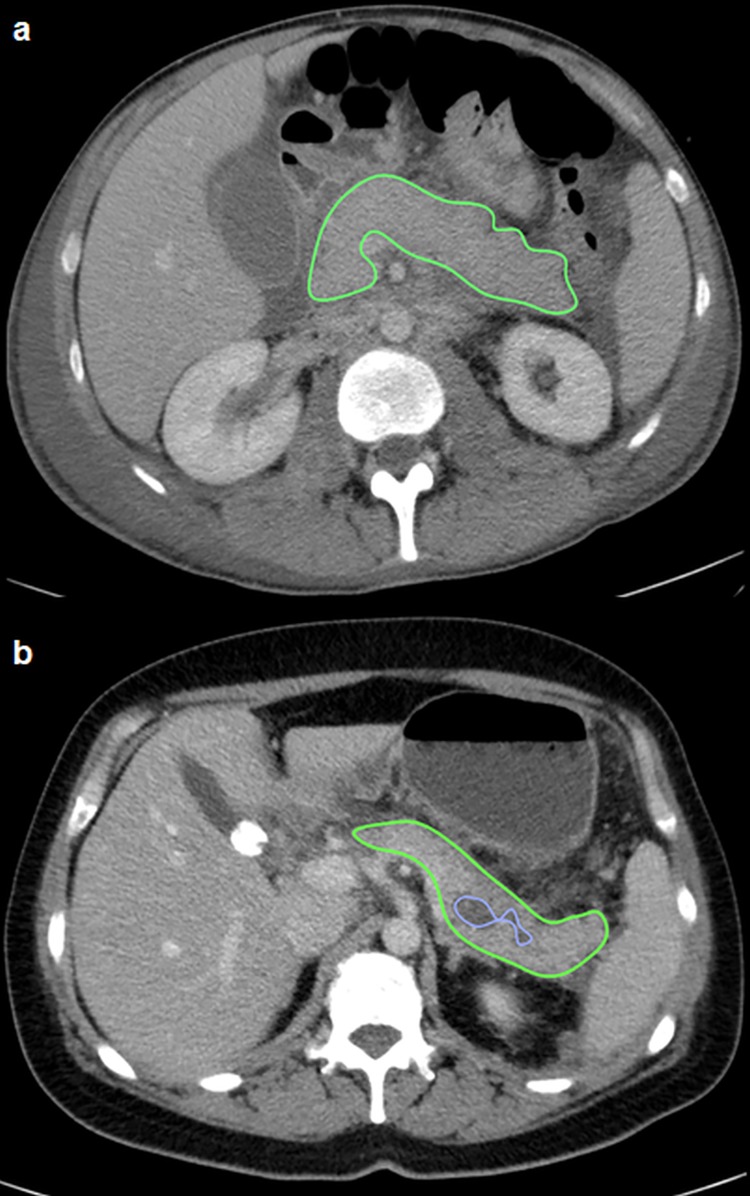
**Volumetry of the pancreatic parenchyma of a 56-year old male patient with a interstitial edematous pancreatitis (a) and a volumetry of the pancreatic parenchyma and intrapancreatic necrosis in a 48-year old male patient with necrotizing pancreatitis (b).** The pancreatic tissue border is delineated by green colour (a+b), whereas the intrapancreatic necrosis is delineated by blue colour (b).

All volumetric assessments were performed by the most experienced observer with 34 years of experience in abdominal imaging (AS) on axial planes by semi-automated software (Osirix Lite, 8.5.1, Pixmeo, Suisse). The surface edge of the pancreas was contoured by a region of interest (ROI) in each slice using a window setting of 60–340 HU and manual correction of cases with misdeliniation of the pancreatic tissue borders was performed (Fig 1). In patients with interstitial edematous pancreatitis, the total pancreas volume was assessed, while the intraparenchymal necrosis and the total pancreas volume were calculated in patients with necrotizing pancreatitis. In case of combined extra- and intrapancreatic necrosis, additional coronal and sagittal reformations of the CT studies were evaluated prior to performing the volumetry to ensure an accurately delineation of the pancreatic surface borders. The volume of the vital pancreatic tissue was defined as the difference between the total pancreas volume and the intraparenchymal necrosis volume.

### Clinical parameters

Venous blood samples were taken from every patient on admission (IQR 0–5 days) prior to the CT examination. A serum lipase level of ≥180 U/L on admission defined a threefold increase of the norm and indicated the presence of AP according to the revised Atlanta classification [10]. In addition, C-reactive protein at 48 h after hospital admission (CRP48) was measured in all patients as one of the most useful and simply accessible single clinical predictive parameter for severe acute pancreatitis [27]. A CRP48 cutoff of >190 mg/dl was defined as the most accurate to predict severe disease, as previously demonstrated [28,29]. In 209 out of 225 patients (93%) including 158 patients (95% of 167 patients) with SAP and 51 patients (88% of 58 patients) with RAP, the modified Marshall score as a hallmark of organ dysfunction could been calculated on admission after assessment of the cardiovascular (systolic blood pressure in mmHg), renal (serum creatinine in mg/dl), and the respiratory system (PaO_2_/FiO_2_ ratio) parameters. A score of ≥2 points in any of the abovementioned organ systems defined organ failure, according to the revised Atlanta classification [10]. In all 209 patients, the score was re-calculated after 48hours to assess for a persistent organ failure. For further statistical analysis and correlation of the abovementioned clinical parameters with the prognosis, we considered only patients with persistent organ failure. In addition, in case of organ dysfunction (score ≥2 points), the total sum of the Marshall score (2–16 points in all organ systems) was calculated in each patient for correlation with the total hospitalization duration.

### Clinical outcome

Outcome parameters were collected from the hospital clinical database and included the total duration of hospitalization (in days), the duration of hospitalization on the intensive care unit (in days), need for intervention (CT-guided percutaneous drainage of pancreatic fluid or fat necrosis, surgical necrosectomy, or both), and the mortality rates over a median follow-up time of 8.3 months (IQR 6–10 months) depending on the mortality date or the last notification in the hospital clinical database. Additionally, the mortality causes were subdivided into pancreatitis-related mortality and mortality due to other reasons.

### Statistical analysis

Categorical variables are given as frequencies and percentages. Continuous variables are given as mean and standard deviation in normally distributed data or as median and interquartile range (IQR) otherwise. Normality of continuous data was assessed by the Shapiro-Wilk test. Comparison of continuous data between both groups was performed by t-test in normally distributed data or by Mann-Whitney U test otherwise. Proportions from two independent samples were compared by Chi-squared test or Fischer’s exact test, where appropriate. For prediction of mortality and the need for intervention, all parameters with p<0.05 on univariate logistic regression were entered into a multivariate logistic regression model using stepwise backward selection (p = 0.1) to receive the final prediction model. The correlation between the intrapancreatic necrosis volume/percentage, and hospital duration was analyzed by the Spearman rank correlation coefficient (Spearman’s rho) due to the non-Gaussian distribution of these parameters. Survival analysis was performed by a Kaplan-Meier survival plot with corresponding logrank test.

Interobserver agreement between the two observers was assessed by intraclass correlation coefficient (ICC) analyses. An ICC value of >0.81 was considered as excellent agreement, ICC 0.61–0.80 as substantial agreement, ICC 0.41–0.60 as moderate agreement, ICC 0.21–0.40 as fair agreement, and ICC < 0.20 as slight agreement. Statistical significance was regarded for 2-tailed p values <0.05. Data collection and statistical calculations were performed using SPSS Statistics 22 software (IBM Inc. SPSS Statistics, Chicago, IL) and MedCalc 15.8 (MedCalc Software, Ostend, Belgium).

## Results

### Patient characteristics

The general characteristics of the investigated study group are presented in Table 1. 167 patients (74%) of all patients had an initial attack of acute pancreatitis (SAP) while 58 patients (26%) had recurrent attacks (range 2–6 attacks) of acute pancreatitis (RAP).

**Table 1:** Basis characteristics of patients with diagnosis of acute pancreatitis.

	Total number of patients	SAP	RAP	P-value
	n = 225 (100%)	n = 167 (74%)	n = 58 (26%)	
**Clinical findings**				
Male, n (%)	150 (67)	105 (63)	45 (78)	**0.04**
Age, years	56±15	57 ±15	54 ±13	0.09
Serum lipase on admission, U/L	364(182–1770)	643(202–2113)	192(90–378)	**<0.001**
Systolic blood pressure, mmHg	139±27	138±25	144±32	0.23
Diastolic blood pressure, mmHg	81±15	80±14	84±18	0.14
Serum creatinine, mg/dl	0.9 (0.7–1.1)	0.9 (0.7–1.2)	0.8 (0.7–1.0)	0.052
PaO_2_/FiO_2_	243 (157–367)	258 (168–389)	200 (139–289)	**0.02**
Modified Marshall score ≥2#	46/209 (22)	36/158 (23)	10/51(20)	0.19
CRP48 (mg/dl)	158(76–240)	176(90–256)	108(67–177)	**0.007**
CRP>190mg/dl, n (%)	81 (36)	68 (41)	13 (22)	**0.01**
**Etiology, n (%)**				
Alcohol	62 (28)	25 (15)	37 (64)	**<0.001**
Biliary obstruction	79 (35)	65 (39)	14 (24)	**0.04**
Post-endoscopic retrograde cholangiopancreaticography	23 (10)	22 (13)	1 (2)	**0.01**
Post-surgery*	17 (8)	16 (10)	1 (2)	**0.02**
Toxic (valproat, ciprofloxacin)	8 (4)	7 (4)	1 (2)	0.38
Malignancy (Adenocarcinoma)	1 (0.4)	1 (0.6)	0 (0)	0.56
Other causes§	9 (4)	8 (5)	1 (2)	0.31
Unclear	26 (12)	23 (14)	3 (5)	0.08
**CECT findings**				
Pleural effusion, n (%)	131 (58)	106 (63)	25 (43)	**0.007**
**Morphology, n (%)**				
Acute interstitial pancreatitis	109 (48)	79 (47)	30 (52)	0.56
Acute necrotizing pancreatitis	116 (52)	88 (53)	28 (48)	0.56
**Pancreatic necrosis, n (%)**				
Parenchymal necrosis	33 (15)	29 (33)	4 (14)	0.06
Combined parenchymal-peripancreatic necrosis	77 (34)	56 (64)	21 (75)	0.27
Peripancreatic necrosis	6 (3)	3 (3)	3 (11)	0.13
**Visual assessment of parenchymal necrosis**				
<30%	56 (48)	42 (48)	14 (50)	0.83
30–50%	24 (21)	18 (20)	6 (21)	0.91
>50%	36 (31)	28 (32)	8 (29)	0.75
**CT-based severity scores**				
CTSI	4 (3–8)	4 (3–7)	4 (1–8)	0.26
mCTSI	6 (4–8)	6 (4–8)	6 (2–8)	0.49
EPIC	5 (3–7)	5 (3–7)	4 (2–6)	**0.02**

Data are given as n (%) or mean±SD, median (IQR). #Modified Marshall score was measurable in 209 out of 225 (93%) patients.

*Surgery within the SAP group (n = 16) included resection of tumors of the ampulla of Vater (n = 5), esophagectomy (n = 3), aortic prosthesis (n = 3), colon resection (n = 2), resection of cholangiocarcinoma (n = 2), and pedicle screw instrumentation (n = 1). Surgery within the RAP group (n = 1) included a resection of a tumor of the ampulla of Vater (n = 1). §Other causes within the SAP group (n = 8) included: HIV (n = 1), SLE (n = 1), hypertriglyceridemia (n = 3), hyperlipidemia (n = 2), and a dislocated endobarrier (n = 1). Other causes within the RAP group (n = 1) included pancreas divisum (n = 1).

### Clinical findings

Significantly more male patients developed AP within the RAP group compared with the SAP groups (78% vs. 63%, p<0.05). The assumed etiology of AP differed widely across both investigated groups: Alcohol consumption was the predominant cause of AP in the RAP group compared with the SAP group (64% vs. 15%, p<0.05). In contrast, biliary obstruction, ERCP, and surgery were the major causes of AP in the SAP group and were found significantly more often in the SAP than in the RAP group (p<0.05 for all comparisons of RAP with SAP, Table 1). Thereby, surgery within the SAP group (n = 16) included resection of tumors of the ampulla of Vater (n = 5), esophagectomy (n = 3), aortic prosthesis (n = 3), colon resection (n = 2), resection of cholangiocarcinoma (n = 2), and pedicle screw instrumentation (n = 1). Surgery within the RAP group (n = 1) included a resection of a tumor of the ampulla of Vater (n = 1).

Other causes of AP including HIV (n = 1), SLE (n = 1), hypertriglyceridemia (n = 3), hyperlipidemia (n = 2), and a dislocated endobarrier (n = 1) in the SAP group and a pancreas divisum (n = 1) in the RAP group, as well as the age of patients did not differ significantly among the abovementioned groups.

Median serum lipase on admission, PaO_2_/FiO_2_ ratio, and median CRP48 values were significantly higher in the SAP group compared with the SAP group (serum lipase: 643U/L (SAP) vs. 192U/L (RAP), p<0.001; PaO_2_/FiO_2_: 258 (SAP) vs. 200 (RAP), p = 0.02, CRP48: 176mg/dl (SAP) vs. 108mg/dl (RAP), p<0.05). In accordance, a CRP48 of >190mg/dl as single clinical indicator of a severe disease was significantly increased in the SAP group compared with the RAP group (41% (SAP) vs. 22% (RAP), p<0.05, Table 1).

### Computed tomography findings

The morphologic types of AP were comparable within both groups (acute interstitial AP: 47% (SAP) vs. 52% (RAP), p = ns; necrotizing AP: 53% (SAP) vs. 48% (RAP), p = ns, Table 1). In addition, neither considering the distribution of the necrosis nor in the extent of intrapancreatic necrosis a significant difference between both study groups was found (p = ns).

In contrast, the frequency of pleural effusion varied significantly among both groups. In the SAP group significantly more effusions were observed compared with the RAP (63% (SAP) vs. 43% (RAP), p<0.01). Among the three CT-based severity scores (CTSI, mCTSI, EPIC), EPIC was the only score with a significant difference between both groups (5 (IQR3-7) vs. 4 (IQR2-6), p<0.05).

### Comparison of pancreatic volumes

**Fig 2:**
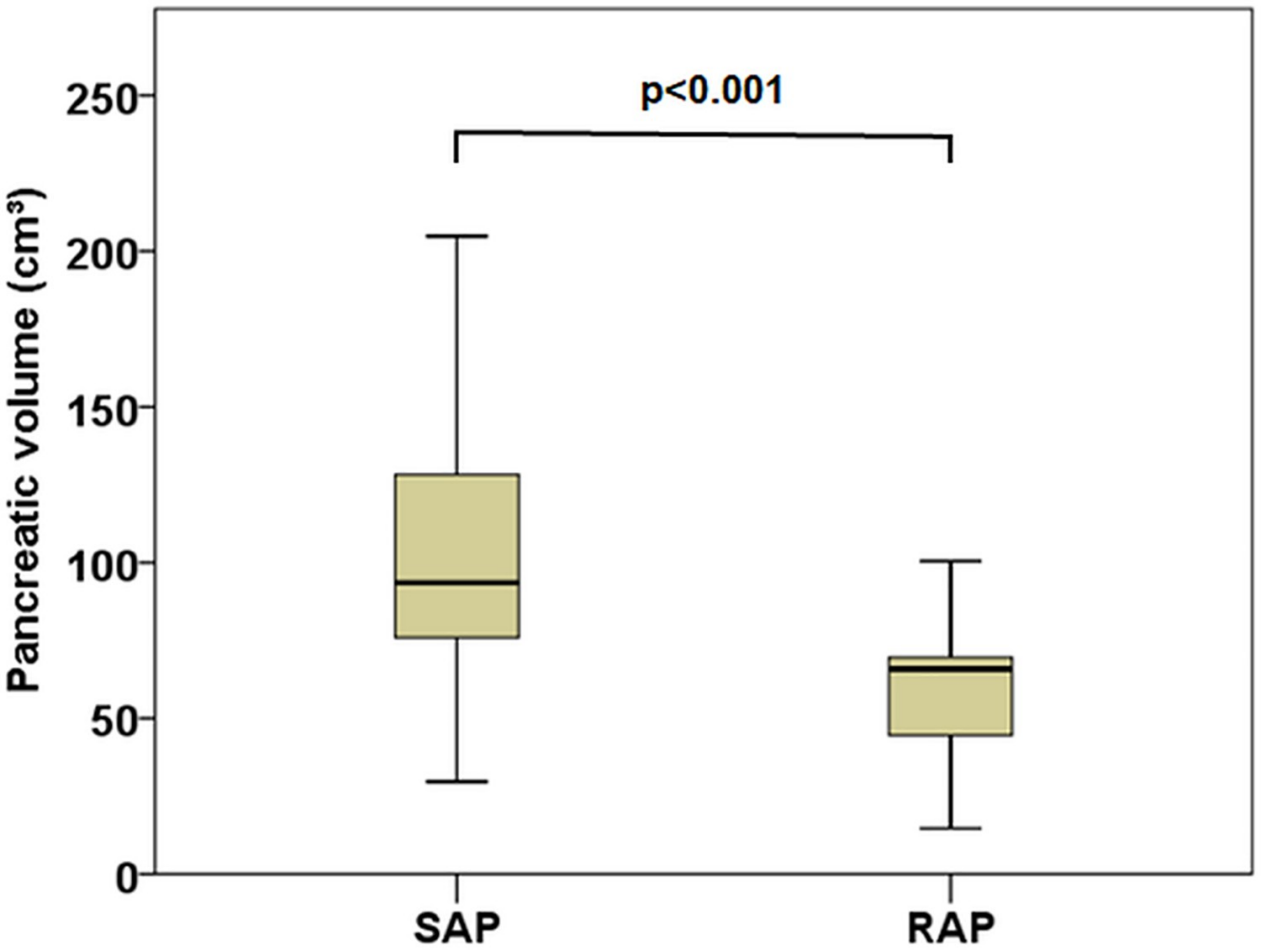
Comparison of the pancreatic volume in patients with interstitial edematous single acute pancreatitis (SAP) and recurrent acute pancreatitis (RAP) using boxplots.

In patients with interstitial acute pancreatitis the total pancreatic volume was significantly larger in the SAP group compared with the RAP (median 94cm^3^; (IQR76-129)/mean 106±45cm^3^; (SAP) vs. median 66cm^3^; (IQR45-70)/mean 69±35cm^3^;, p<0.001, Fig 2).

However, in patients with necrotizing acute pancreatitis the total pancreatic volume did not differ significantly between both groups (SAP: 131 (IQR 94–195) cm^3^; vs. RAP:107 (IQR 69–170) cm^3^;, p = ns). Moreover, the extent of parenchymal necrosis by visual assessment and by semi-automated volumetry was also comparable in both groups (S1 Table, p = ns for all comparisons).

### Clinical outcomes

In both acute interstitial and necrotizing pancreatitis the total duration of hospitalization, the duration of hospitalization on an intensive care unit, and the need for intervention was comparable within the investigated groups (for all p = ns, Table 2). However, a trend for higher duration on an intensive care unit was observed for the SAP group compared with the other two groups (p = 0.07).

**Table 2:** Clinical outcomes.

	SAP	RAP	P-value
**Acute interstitial pancreatitis**	**n = 79**	**n = 30**	
Total duration of hospitalization (d)	13 (8–22)	11 (6–18)	0.13
duration of hospitalization on intensive care unit (d)	0 (0–7)	0 (0)	0.07
Need for intervention,n (%)	8 (10)	2 (7)	0.24
Percutaneous catheter drainage, n (%)	4 (5)	0 (0)	0.21
Surgical necrosectomy, n (%)	1 (1)	1 (3)	0.24
Both, n (%)	3 (4)	1 (3)	0.91
**Acute necrotizing pancreatitis**	**n = 88**	**n = 28**	
Total duration of hospitalization (d)	26 (14–51)	37 (21–55)	0.32
duration of hospitalization on intensive care unit (d)	2 (0–17)	7 (0–22)	0.25
Need for intervention,n (%)	43 (49)	17 (61)	0.32
Percutaneous catheter drainage,n (%)	13 (15)	4 (14)	0.71
Surgical necrosectomy, n (%)	10 (11)	4 (14)	0.70
Both,n (%)	20 (23)	9 (32)	0.61
**Mortality**	**n = 167**	**n = 58**	
Deaths due to MOV after necrotizing pancreatitis, n (%)#	25 (15)	4 (7)	0.11
Other death causes than pancreatitis, n (%)*	4 (2)	3 (5)	0.55
**Time delay between admission and death,(days)**			
Any cause of death	48 (25–94)	24 (18–63)	0.26
Pancreatitis-related death	50 (29–94)	19 (10–44)	0.24

Data are given as % (n) or median (IQR).

#No deaths had occurred due to acute interstitial pancreatitis.

*Other death causes included metastatic Klatskin tumor, mesenteric ischemia, pneumonia with lung edema, cardiogenic shock due to right heart failure, sepsis due to ARDS.

**Fig 3:**
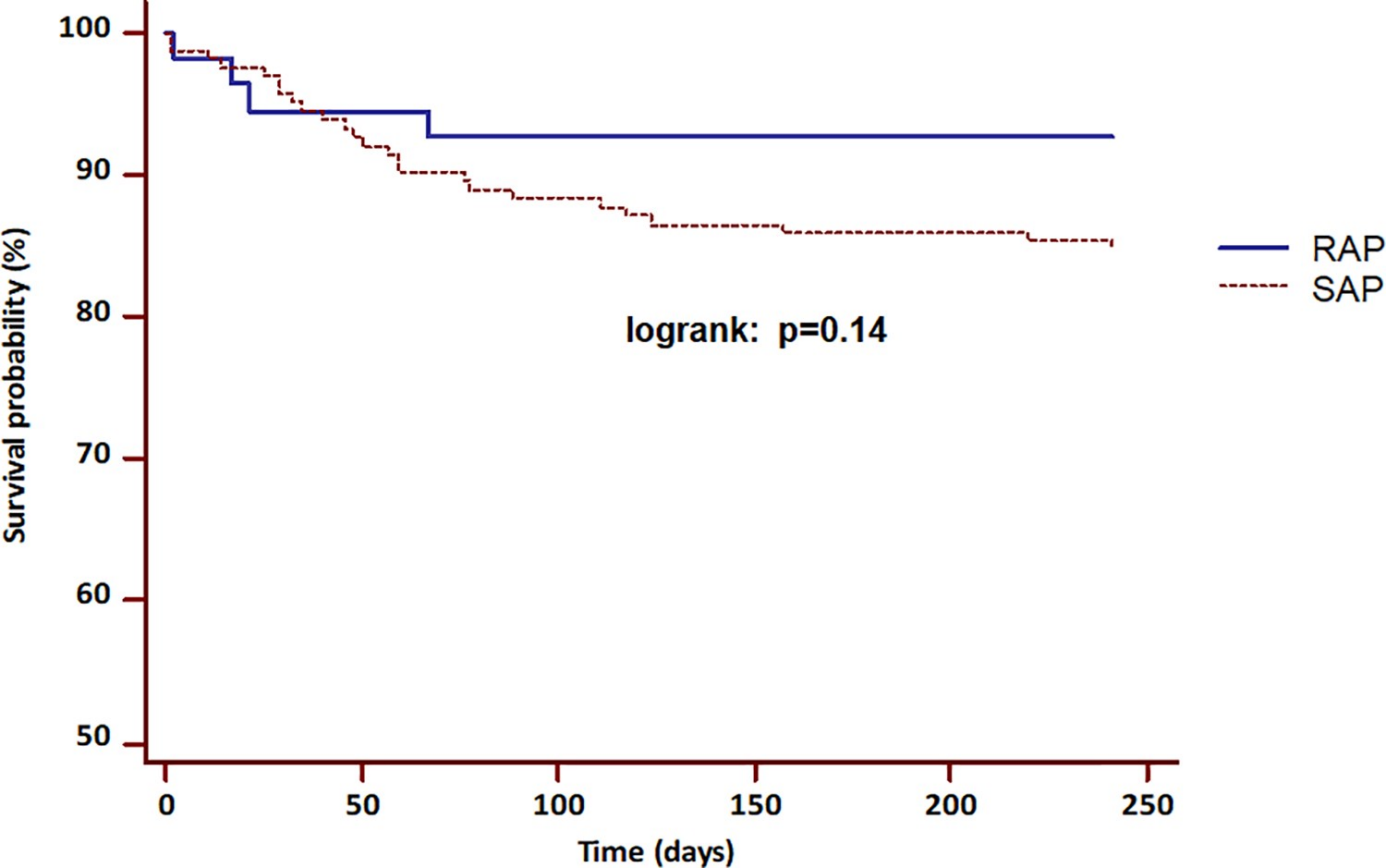
Comparison of two Kaplan-Meier curves of the pancreatitis-related short-term mortality between the RAP and SAP groups. No significant difference of the observed mortality was present between both groups (logrank: p = 0.14).

Regarding the mortality rates after a median follow-up of 8.3 months, no difference in mortality frequency was observed between the SAP and the RAP groups (logrank p = 0.14, Fig 3). None of the patients died as a consequence of an interstitial edematous pancreatitis. In 6 patients (1 belonged to SAP group, 5 belonged to RAP group) an initial interstitial edematous pancreatitis, which was diagnosed on initial CT progressed to a necrotizing pancreatitis in the further course of the disease. The time from admission to death was also comparable between both groups (p = ns for all).

### Clinico-radiological prognostic parameters

In patients with SAP who died due to AP, CRP48 values, presence of pleural effusions, CTSI, mCTSI, EPIC, systolic blood pressure, serum creatinine, and the modified Marshall score were significantly increased compared to those, who survived after AP in univariate analysis (p<0.05 for all. Pancreatic volumes as well as the necrosis volumes were comparable in both groups of SAP patients (p = ns for all).

Patients with SAP, who needed an intervention (necrosectomy, CT-guided drainage, or both), revealed significantly higher extents of intrapancreatic necrosis (p<0.05) as well as increased values of systolic blood pressure (p<0.05), CTSI, mCTSI, and EPIC values (p<0.05 for all scores, Table 3).

**Table 3:** Univariate analysis between clinico-radiological parameters, mortality and need for intervention in patients with single acute pancreatitis (SAP).

*SAP*	Death due to pancreatitis	Survival after pancreatitis	P-value	Need for intervention	No need for intervention	P-value
	*n = 25*	*n = 138*	-	*n = 51*	*n = 116*	-
Lipase	647 (224–2169)	414 (199–1610)	0.14	817(266–2285)	232(157–1531)	0.26
CRP48	213 (182–273)	164 (82–256)	**0.04**	181 (114–292)	166 (71–245)	0.22
Pleural effusion, n (%)	21 (84)	82 (59)	**0.02**	34 (67)	71 (61)	0.50
CTSI	7 (6–9)	4 (2–6)	**<0.001**	8 (5–10)	4 (2–5)	**<0.001**
mCTSI	9 (6–10)	6 (3–8)	**<0.001**	9 (6–10)	6 (3–7)	**<0.001**
EPIC	7 (5–7)	5 (3–6)	**<0.001**	6 (5–7)	4 (3–6)	**<0.001**
Total pancreatic volume (ml)	114 (77–225)	92 (60–130)	0.06	97 (72–129)	86 (44–142)	0.41
**Modified Marshall score**	**n = 24**	**n = 131**	-	**n = 48**	**n = 110**	-
Systolic blood pressure, mmHg	128±20	140±26	**0.04**	130±19	141±27	**0.005**
Serum creatinine, mg/dl	1.5 (0.9–2.1)	0.9 (0.7–1.1)	**0.004**	1.0 (0.7–1.6)	0.9 (0.8–1.2)	0.40
PaO_2_/FiO_2_	252 (157–369)	322 (195–462)	0.35	243 (156–367)	331 (195–481)	
Modified Marshall score ≥2	13 (54)	21 (16)	**<0.001**	11 (23)	25 (23)	0.86
***Volumetry in*** ***necrotizing pancreatitis***	n = 21*	n = 63*	-	***n = 43***	***n = 42***	-
Intrapancreatic necrosis (ml)	52 (19–134)	25 (7–90)	0.22	64 (16–167)	22 (5–45)	**0.001**
vital pancreatic volume (ml)	55 (31–117)	84 (47–119)	0.38	55 (33–108)	96 (54–121)	0.07
necrosis %	39 (20–78)	25 (7–63)	0.16	40 (17–76)	17 (42)	**0.005**

* 1 patient developed necrotizing pancreatitis after initial CT; 3 other patients had extrapancreatic necrosis and were excluded from volumetry assessment

In the RAP group, systolic blood pressure was the only parameter, which differed significantly between those patients, who died and who survived and those who required and did not require an intervention (p<0.05 for all, Table 4).

**Table 4:** Univariate analysis between clinico-radiological parameters, mortality and need for intervention in patients with recurrent acute pancreatitis (RAP).

*RAP*	Death due to pancreatitis	Survival after pancreatitis	P-value	Need for intervention	No need for intervention	P-value
	*n = 4*	*n = 51*	-	*n = 19*	*n = 39*	-
Lipase	195 (91–557)	169 (87–239)	0.47	188 (90–365)	155 (90–214)	0.29
CRP48	115 (67–180)	96 (75–114)	0.46	164 (107–203)	122 (97–231)	0.67
Pleural effusion, n (%)	3 (75)	23 (45)	0.25	11 (58)	9 (41)	0.28
CTSI	7 (5–8)	4 (3–7)	0.16	5 (4–8)	4 (2–7)	0.42
mCTSI	7 (5–9)	6 (4–8)	0.49	7 (6–9)	6 (3–8)	0.22
EPIC	5 (3–5)	4 (2–6)	0.87	5 (4–6)	4 (2–6)	0.45
Total pancreatic volume (ml)	77 (49–111)	67 (51–140)	0.82	77 (57–110)	63 (44–115)	0.27
**Modified Marshall score**	**n = 4**	**n = 44**	-	**n = 16**	**n = 35**	-
Systolic blood pressure, mmHg	108±11	148±31	**<0.001**	131±19	150±35	**0.02**
Serum creatinine, mg/dl	1.2 (0.9–2.0)	0.9 (0.7–1.0)	0.32	1.0 (0.7–1.1)	0.8 (0.7–1.0)	0.34
PaO_2_/FiO_2_	119 (81–267)	203 (148–290)	0.17	193 (143–281)	238 (132–367)	0.59
Modified Marshall score ≥2 §	1 (25)	9 (20)	1.0	5 (31)	5 (14)	0.25
***Volumetry in*** ***necrotizing pancreatitis***	***n = 4***	***n = 16#***	-	***n = 14‘***	***n = 12***	-
Intrapancreatic necrosis (ml)	61 (21–101)	35 (19–65)	0.67	31 (17–64)	33 (15–60)	0.96
vital pancreatic volume (ml)	140 (72–209)	80 (57–119)	0.40	66 (45–109)	64 (42–128)	0.64
necrosis %	28 (23–33)	25 (13–49)	0.78	26 (22–43)	24 (16–54)	0.91

# 5 patients developed necrotizing AP after initial CT and other 3 patients had extrapancreatic necrosis and were excluded from volumetry assessment.

‘ 2 patients had extrapancreatic necrosis and were excluded from volumetry measurements.

§Fisher’s exact test

In multivariate logistic regression analysis, the modified Marshall score (β: 1.79, Wald statistic: 11.94, p<0.001) and mCTSI (β: 0.40, Wald statistic: 12.65, p<0.001).)were the only two predictors for mortality due to pancreatitis in the SAP group) In contrast, in patients with RAP, only systolic blood pressure showed a weak negative predictive value for mortality due to pancreatitis (β: -0.05, Wald statistic: 4.71, p = 0.03, Table 5).

**Table 5:** Logistic regression analysis of clinico-radiological predictor variables for mortality due to pancreatitis and the need for intervention.

***Variable***	**Mortality due to pancreatitis**			
	***β***	***Wald***	***p-value***	***Odds ratio (95%CI)***
**SAP**				
Modified Marshall score	1.79	11.94	<0.001	6.01 (2.17–6.62)
mCTSI	0.40	12.65	<0.001	1.49 (1.20–1.86)
**RAP**				
Systolic blood pressure	-0.05	4.71	0.03	0.95 (0.91–0.99)
***Variable***	**Need for intervention**			
	***β***	***Wald***	***p-value***	***Odds ratio (95%CI)***
**SAP**				
CTSI	0.46	33.10	<0.001	1.58 (1.35–1.85)
Intrapancreatic necrosis (ml)	0.17	4.42	0.03	1.18 (1.01–1.39)
**RAP**				
Systolic blood pressure	-0.02	3.48	0.049	0.98 (0.96–1.00)

CTSI: CT Severity Index; mCTSI: Modified CT Severity Index; SAP: single acute pancreatitis; RAP: recurrent acute pancreatitis

**Fig 4:**
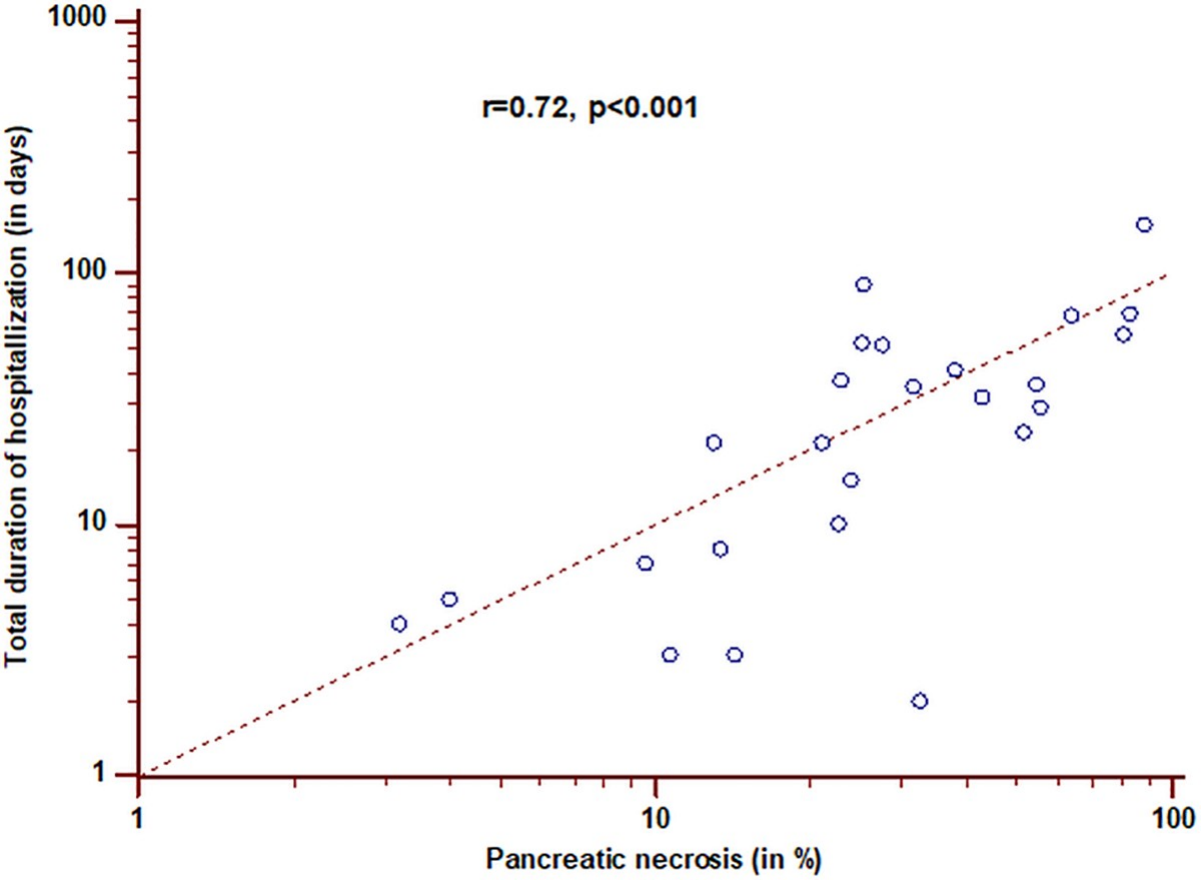
Correlation of the percentage extent of pancreatic necrosis with the total duration of hospitalization among patients with necrotizing RAP.

Regarding the predictive parameters for the need of intervention by multivariate logistic regression analysis, the CTSI (β-coefficient: 0.46, Wald statistic: 33.10, p<0.001) and the extent of intrapancreatic necrosis (β-coefficient: 0.17, Wald statistic: 4.42, p = 0.03) were the only two predictors in the SAP group. However, in the RAP cohort, again only the systolic blood pressure revealed a weak negative predictive value for an intervention (β-coefficient: -0.02, Wald statistic: 3.48, p = 0.048) No significant correlation was found between the total sum of the Marshall score, the CT-based scores, and the total hospitalization duration in patients with SAP or RAP (p = ns for all comparisons) However, the total hospitalization duration correlated negatively with systolic blood pressure (Spearman’s rho = -0.21, p = 0.010) and positively with serum creatinine (Spearman’s rho = 0.20, p = 0.010), but not with the PaO_2_/FiO_2_ ratio (Spearman’s rho = 0.13, p = ns) within the SAP group The only significant correlation between the percentage extent of pancreatic necrosis and the total duration of the hospitalization was found in patients with necrotizing RAP (r = 0.72, p<0.001, Fig 4),in contrast to patients with necrotizing SAP (r = 0.21, p = ns).

Moreover, the total hospitalization duration on ICU in SAP patients correlated weakly with the Marshall sum score (Spearman’s rho = 0.36, p = 0.03). However, no significant correlations were observed between total hospitalization duration on ICU,and any clinic-radiological parameters in RAP patients (p = ns for all correlations).

### Interobserver agreement

With regard to the interobserver agreement for the applied CT severity scores for pancreatic (CTSI, mCTSI) and extrapancreatic inflammatory changes (mCTSI, EPIC), our data revealed an excellent agreement in patients with any type of AP with ICC values of 0.81–0.83. Substantial to excellent agreement results were found in patients with acute interstitial edematous pancreatitis with ICC values of 0.80–0.84 and necrotizing pancreatitis with ICC values of 0.79–0.83. Detailed results from interobserver agreements are demonstrated in Table 6.

**Table 6:** Interclass correlation coefficients (ICC) between two observers regarding 3 CT severity scores applied to patients with any type of acute pancreatitis and separated according to the morphologic type of acute pancreatitis.

	patients with any type of acute pancreatitis n = 225	Interstitial edematous pancreatitis n = 109	Necrotizing pancreatitis n = 116
**CTSI**	0.82 (0.77–0.86)	0.81 (0.72–0.86)	0.79 (0.69–0.87)
**mCTSI**	0.83 (0.79–0.87)	0.84 (0.76–0.89)	0.83 (0.75–0.88)
**EPIC**	0.81 (0.72–0.86)	0.80 (0.71–0.85)	0.80 (0.72–0.85)

Data are ICC values (95% Confidence interval)

## Discussion

In the present study we assessed different clinico-radiological parameters to compare patients with SAP and RAP and to find prognostic parameters for short-term mortality, need for intervention and hospitalization duration in both groups. Moreover, we evaluated the additional value of volumetric measurements of pancreatic parenchyma and intrapancreatic necrosis for short-term prognosis in both groups of AP.

The first major finding was a significantly increased total pancreatic volume in patients with interstitial edematous SAP compared to those with RAP. This result is in line with the necrosis-fibrosis hypothesis [4], according to which the inflammatory response to an AP episode leads to collagen deposition in the affected periductal areas with progressive obstruction of the acinar cell complex and resulting acinar cell atrophy and secondary stone formation. Consequently, this finding underlines the current opinion of RAP acting as link between acute and chronic pancreatitis [30,31], where the latter is demarcated by visual pancreatic atrophy and/or parenchymal and intraducal calculi formation as a long-term consequence of the abovementioned processes [32–35]. Moreover, the few published studies of the normal pancreas volumes on CT revealed mean pancreas volumes of 72.4 ±25.8cm^3^; [36] and 79.2±24.1cm^3^; [37] in both sexes, which are in between our mean pancreatic volumes of 69±35cm^3^; in patients with interstitial RAP and 106±45cm^3^; in patients with interstitial SAP. Moreover, former studies demonstrated stable volumetric values in adults between 20–60 years [36] or even no correlation with age [37]. However, in both studies the total pancreatic volumes significantly correlated with gender with larger volumes in men [36,37]. The assessment of total pancreas volume might enable a more precise, quantitative estimation of the degree of pancreas atrophy and replace the solely visual assessment of pancreatic parenchyma or older volume estimation methods based on the pancreatic duct caliber [38], which may be prone to errors, e.g. in case of acute pancreatic duct dilatation due to a stone or a poor delineation of the pancreatic duct.

Interestingly, the volumetric extent of intrapancreatic necrosis was comparable in patients with necrotizing AP in both SAP and RAP groups, whereas median serum lipase levels were only slightly above a threefold increase of the norm in the RAP group and differed significantly from median lipase levels in the SAP group. This is supported by previous results [39,40], indicating an ongoing decline of pancreatic tissue and lipase levels in patients with RAP [40], and possibly ending in normal lipase levels in chronic pancreatitis [39].

The second major finding refers to the fact, that our investigated outcome parameters including the mortality rate, the need for intervention, and the duration of hospitalization were comparable in the SAP and RAP groups. The mortality results are in concordance with a multi-center study [41], where all patients who died had necrotizing pancreatitis and similar mortality rates were observed between patients with SAP and RAP. In detail, similar mortality rates of 5.9% were observed in patients with RAP compared with our results of 7%. However, the mortality of 15% in our SAP group was higher compared with the reported SAP mortality of 8.5% [41], which might be explained by the much older study population compared with the reported data (mean age 56 years (present study) vs. 43 years (multicenter-study)) and possibly higher rates of potentially life-threatening comorbidities, as previously reported [42]. The comparable number of interventions and duration of hospitalization in both groups may be explained by the homogeneous allocation of interstitial edematous and necrotizing AP among both groups (necrotizing AP: 53% (SAP) vs. 48% (RAP), p = 0.56).

The third major finding contains the variety of the prognostic parameters for each outcome parameter in both groups. In patients with SAP, the modified Marshall score was the best predictor for pancreatitis-related short-term mortality followed by mCTSI, which correlates with previous findings [10,24,43,44]. CTSI was the best predictor for the necessity of intervention, followed by the volume of intrapancreatic necrosis in necrotizing SAP. These results are also supported by previous works [44,45] and may be explained by the fact, that CTSI also includes the evaluation of peripancreatic fluid collections, which might be treated by percutaneous drainage placement. In contrast, the value of the intrapancreatic necrosis volume for intervention prediction is probably lower due to its limitation to the intrapancreatic necrosis areas, which are reached less easily by percutaneous drainage than extrapancreatic necrosis collections.

Surprisingly, in patients with RAPonly the systolic blood pressure revealed a small prognostic value for the short-term mortality and the need for intervention with lower systolic blood pressure values increasing the risk of mortality and need for intervention. This finding may be explained by the well-known hypovolemic status of patients with acute pancreatitis, which is related to its severity [46]. Hypolovemia may be aggravated in patients with RAP due to a high number of alcoholics within this group [6–8,14]. Moreover, alcohol is known to impair homeostatic counter regulation after volume loss and worsen the outcome in patients with hemorrhagic shock [47–48]. However, the percentage extent of pancreatic necrosis in patients with necrotizing RAP revealed a strong correlation with the total duration of hospitalization, indicating its prognostic, but limited value due to possible confounders like age and other comorbidities. These findings may indicate a failure of many of the investigated and currently used clinico-radiological parameters to assess discrete early changes in RAP with sufficiently high sensitivity and specificity, which are relevant for outcome prediction. Secretin-enhanced endoscopic ultrasonography and MRCP were shown to have high diagnostic yields for subtle pancreatic duct and/or parenchymal abnormalities [49,50] and may potentially represent better outcome predictors in RAP, which should be assessed in upcoming studies. With the advent of new fully automated deep learning based segmentation algorithms for pancreas segmentation on abdominal CT [51–53], more research is necessary to evaluate the full prognostic value of both, the intra- and extrapancreatic extent of pancreatic necrosis in SAP and RAP.

The major limitation of the present study is its retrospective design. However, to our knowledge, this is the first study, which evaluated the diagnostic and prognostic value of total pancreatic and necrosis volume in patients with SAP and RAP. Another possible limitation refers to the measurement of solely intrapancreatic necrosis volume in contrast to Meyrignac et al.[17]. However, extrapancreatic necrosis assessment is currently limited by its low reproducibility and the large amount of time, which may be reduced by fully automated pancreatic necrosis segmentation models. Furthermore, clinical severity scores like APACHE-II or BISAP [54,55] were not assessed due to its complicity and/or missing parameters for calculation with regard to the retrospective study design.Instead of these scores, we assessed the persistent organ dysfunction by the simpler modified Marshall score, as proposed by the revised Atlanta classification [10] and correlated that score with different prognostic parameters.

## Conclusion

Our results indicate the potential value of total pancreas volume to further distinguish interstitial RAP from SAP beyond patients’ history. Despite comparable outcome (short-term mortality, need for intervention, and hospitalization duration) in both groups, only two parameters were predictive in RAP. Intrapancreatic necrosis strongly correlated with total hospitalization duration, whereas systolic blood pressure was a weak prognostic factor for required interventions and the short-term mortality. In SAP, only the modified Marshall score and mCTSI were predictive for short-term mortality, while CTSI and the extent of intrapancreatic necrosis revealed prognostic value for the required interventions.

## Supporting information

S1 Table Comparison of the visual and volumetric extent of parenchymal pancreatic necrosis in necrotizing pancreatitis. (DOCX)

## Abbreviations

AP: Acute pancreatitis
CECT: Contrast-enhanced computed tomography
CP: Chronic pancreatiis
CTSI: CT Severity Index
EN: Extrapancreatic necrosis
EPIC: Extrapancreatic inflammation on CT score
HU: Hounsfield Units
IQR: Interquartile range
ICC: Interclass correlation
mCTSI: Modified CT Severity Index
SAP: Single acute pancreatitis
RAP: Recurrent acute pancreatitis
RIS: Radiological information system
ROI: Region of interest
