# Supplementary material for: Clinico-radiological comparison and short-term prognosis of single acute pancreatitis and recurrent acute pancreatitis including pancreatic volumetry
Source: PLoS One. 2018 Oct 25;13(10):e0206062. doi: 10.1371/journal.pone.0206062 (PMC6201910; doi:10.1371/journal.pone.0206062)
Supplement: S1 Table — (DOCX) [file pone.0206062.s001.docx]

# Supporting information

**S1 Table.** Comparison of the visual and volumetric extent of parenchymal pancreatic necrosis in necrotizing pancreatitis

|  | **SAP**  **N=85*** | **RAP**  **N=25*** | **p-value** |
| --- | --- | --- | --- |
| **Visual extent of**  **parenchymal necrosis** | | |  |
| <30%  30-50%  >50% | 42 (48)  18 (20)  28 (32) | 14 (50)  6 (21)  8 (29) | 0.83  0.91  0.75 |
| **Volumetric extent of**  **parenchymal necrosis** | | |  |
| Intrapancreatic necrosis (ml) | 28 (8-91) | 33 (16-61) | 0.96 |
| healthy pancreas volume (ml) | 76 (45-117) | 64 (45-117) | 0.79 |
| necrosis % | 29 (8-65) | 25 (19-53) | 0.91 |

*3 patients had extrapancreatic necrosis in both groups and were excluded from volumetric assessment
